# Community Composition and Transcriptional Activity of Ammonia-Oxidizing Prokaryotes of Seagrass *Thalassia hemprichii* in Coral Reef Ecosystems

**DOI:** 10.3389/fmicb.2018.00007

**Published:** 2018-01-25

**Authors:** Juan Ling, Xiancheng Lin, Yanying Zhang, Weiguo Zhou, Qingsong Yang, Liyun Lin, Siquan Zeng, Ying Zhang, Cong Wang, Manzoor Ahmad, Lijuan Long, Junde Dong

**Affiliations:** ^1^CAS Key Laboratory of Tropical Marine Bio-resources and Ecology, Guangdong Provincial Key Laboratory of Applied Marine Biology, South China Sea Institute of Oceanology, Chinese Academy of Sciences, Guangzhou, China; ^2^University of Chinese Academy of Sciences, Beijing, China; ^3^Tropical Marine Biological Research Station in Hainan, South China Sea Institute of Oceanology, Chinese Academy of Sciences, Sanya, China

**Keywords:** seagrass, ammonia-oxidizing archaea and bacteria, ammonia monooxygenase subunit A (*amo*A), cDNA, community structure, coral reef ecosystems

## Abstract

Seagrasses in coral reef ecosystems play important ecological roles by enhancing coral reef resilience under ocean acidification. However, seagrass primary productivity is typically constrained by limited nitrogen availability. Ammonia oxidation is an important process conducted by ammonia-oxidizing archaea (AOA) and bacteria (AOB), yet little information is available concerning the community structure and potential activity of seagrass AOA and AOB. Therefore, this study investigated the variations in the abundance, diversity and transcriptional activity of AOA and AOB at the DNA and transcript level from four sample types: the leaf, root, rhizosphere sediment and bulk sediment of seagrass *Thalassia hemprichii* in three coral reef ecosystems. DNA and complementary DNA (cDNA) were used to prepare clone libraries and DNA and cDNA quantitative PCR (*q*PCR) assays, targeting the ammonia monooxygenase-subunit (*amo*A) genes as biomarkers. Our results indicated that the closest relatives of the obtained archaeal and bacterial *amo*A gene sequences recovered from DNA and cDNA libraries mainly originated from the marine environment. Moreover, all the obtained AOB sequences belong to the *Nitrosomonadales* cluster. Nearly all the AOA communities exhibited higher diversity than the AOB communities at the DNA level, but the *q*PCR data demonstrated that the abundances of AOB communities were higher than that of AOA communities based on both DNA and RNA transcripts. Collectively, most of the samples shared greater community composition similarity with samples from the same location rather than sample type. Furthermore, the abundance of archaeal *amo*A gene in rhizosphere sediments showed significant relationships with the ammonium concentration of sediments and the nitrogen content of plant tissue (leaf and root) at the DNA level (*P* < 0.05). Conversely, no such relationships were found for the AOB communities. This work provides new insight into the nitrogen cycle, particularly nitrification of seagrass meadows in coral reef ecosystems.

## Introduction

Many investigations into the effect of ocean acidification (OA) on coral reefs have been conducted (Andersson and Gledhill, 2013), and results indicate that that marine organisms which inhabit the carbonate structures of coral reefs are important and sensitive to OA (Larkum et al., 2006). Albright et al. (2016) and Lough (2016) found that changes to pH in the seawater surrounding natural coral reefs in the southern Great Barrier Reef can significantly affect calcification rates, suggesting that OA may already be altering the growth of coral reefs. By decreasing the ocean pH, OA can affect the calcifying rates of calcifying creatures such as coralline algae with carbonate skeletons (Hendriks et al., 2014; Albright et al., 2016). However, seagrasses can increase the pH of the ambient environment via high photosynthesis rates (Lai et al., 2013; Hendriks et al., 2014). Moreover, Lamb et al. (2017) found that seagrass in coral reef ecosystems can reduce disease levels twofold in comparison with the corals located adjacent to seagrass meadows and corals at paired sites without seagrass.

Seagrass is highly productive and of great ecological importance in the marine environment. For instance, it can provide food, nursery and breeding habitats for other marine organisms inhabiting the ecosystem and nutrients for coral reefs. Nonetheless, available nitrogen is usually the main factor limiting the primary productivity of seagrass because coral reef ecosystems are largely oligotrophic. Nitrification is a key process in the nitrogen cycle in the marine environment. The first and rate-limiting step of nitrification is performed by ammonia-oxidizing archaea (AOA) and ammonia-oxidizing bacteria (AOB), both of which are ammonia oxidizers and responsible for converting ammonia to nitrite. However, these microbes have different phylogenetic and physiological features, resulting in significant variations in their abundance, diversity and activity under different environmental conditions. For example, AOA can adapt to a variety of habitats and account for a large proportion of marine and estuary communities (Dang et al., 2008, 2010a,b, 2013; Boyd et al., 2011; Cao et al., 2012; Rusch and Gaidos, 2013). However, Di et al. (2009), Wu et al. (2011), Zhang et al. (2014), Zheng et al. (2014) reported that AOB might play a more significant role in the ammonia oxidation process under certain conditions. Consequently, the relative contribution of AOA and AOB to ammonia oxidation is still in debate.

Zhang J. et al. (2015) suggested that the presence of certain freshwater plants, such as *Iris pseudacorus*, *Thalia dealbata*, and *Typha orientalis L.*, affected the ecological characteristics of AOA and AOB significantly by increasing the abundance of ammonia oxidizers in the rhizosphere sediments. For the marine environment, investigations of Moin et al. (2009) and Chen and Gu (2017) revealed that the presence of *Spartina alterniflora* or *S. patens* and mangrove roots had a strong influence on the diversity and abundance of AOA and AOB in the coastal area and mangrove ecosystems, respectively. Furthermore, investigations of the ammonia-oxidizing prokaryotes have been conducted extensively in many different marine environments including the Changjiang Estuary, the Jiaozhou Bay, the tropical West Pacific Continental Margin, the Okhotsk Sea, the Sargasso Sea, the Northern South China Sea (Mincer et al., 2007; Nakagawa et al., 2007; Dang et al., 2013; Li and Gu, 2013; Newell et al., 2013; Lipsewers et al., 2014; Cao et al., 2015; Yu et al., 2016).

Regardless, almost all the above-mentioned investigations focused on the seawater and sediment using only DNA as a proxy to assess AOA and/or AOB. Few investigations have focused on ammonia-oxidizing prokaryotes in the aquatic benthic flora, particularly for the seagrass ecosystem (Zhao et al., 2014; Bernhard et al., 2016; Frame et al., 2016; Huang et al., 2016; Mejia et al., 2016; Ettinger et al., 2017). Not to mention the studies of AOA and AOB of seagrass in coral reef ecosystems aimed at revealing the transcriptional activity of relevant functional groups in their natural physiological state. Accordingly, in this study, we prepared archaeal and bacterial *amo*A gene DNA and cDNA libraries and performed reverse-transcription polymerase chain reaction (PCR) and real-time quantitative PCR (qPCR) to examine the community abundance, diversity and transcriptional activity of ammonia-oxidizing prokaryotes of the seagrass *Thalassia hemprichii* in the Luhuitou fringing reef, Sanya Bay and Yongxi Island, Xisha Islands. The aims of our investigation were as follows: (i) to evaluate the abundance and diversity of AOA and AOB communities, (ii) to compare community variations within and among the sample types and locations, and (iii) to analyze the transcriptional activity of AOA and AOB.

## Materials and Methods

### Study Sites and Sampling

Seagrass *T. hemprichii* is one of the most widely distributed seagrass species among tropical southern Indo-Pacific flora, and exists in a monospecific or mixed-species status. The sampling locations were distributed in Sanya Bay (SYT) and Yongxing Island (AT and ST), South China Sea (Supplementary Figure S1). Samples at SYT were collected from the Luhuitou fringing reef (18°12′19″ N, 109°28′27″ E) located in Sanya Bay, Hainan Province. The average atmospheric temperature at this site is 30.74°C, with warm summers (34.75°C) and cold winters (27.20°C). The Luhuitou fringing reef area is under the effect of the northeast monsoon (cold and dry winter and spring) and southwest monsoon (warm and wet summer and autumn) during the East Asian monsoon climate (Wu et al., 2008; Cao et al., 2017). Two other sampling locations, AT (16°50′32″ N, 112°20′41″ E) and ST (16°50′6″ N, 112°22′10″ E), are located on Yongxing Island, which is a reef island formed by the accumulation of white coral skeletal material and shell sand on a reef platform. The annual average temperature on Yongxing Island is 26.5°C. This island is also under the effect of the East Asian monsoon (Shen et al., 2017).

Seagrass meadows in the Luhuitou fringing reef and the Yongxing flat reef are representative of the different styles of seagrass meadows in coral reef ecosystems. *T. hemprichii* appeared at the Luhuitou fringing reef after the coral reef has degraded, and it is the only seagrass species present. In the Yongxing Islands, the seagrass is found in a mixed-species status, with seagrasses *Syringodium isoetifolium*, *Halodule uninervis*, and *Halophila ovalis* at AT, whereas *T. hemprichii* was dominant over seagrass *H. ovalis* at ST. In comparison with the Yongxing flat reef, Luhuitou fringing reef areas show higher nutrient concentration, particularly nitrogen, which was attributed to the increasing anthropogenic activity (Cao et al., 2017).

Samples from the Luhuitou fringing reef and Yongxing flat reef were collected on May 28th and June 1st, 2015, respectively. Sampling was carried out according to the methods of Jensen et al. (2007) at low tide. Plants with surrounding sediment were randomly collected using a spade, and immediately transported in sterilized boxes for subsequent subgrouping in triplicate. Sediment from the plant roots and associated invertebrates from leaves and roots were separated by washing with autoclaved seawater. Bulk sediment was also collected at the same area. All sediment samples were collected in triplicate at each location and thoroughly homogenized using a sterilized spoon. Samples collected from one site were divided into four sections: leaves (L); rhizomes and roots (R); rhizosphere sediment (RS) and bulk sediment (S). All samples for DNA/RNA analysis were stored in sample protectors (TaKaRa, Dalian, China), frozen immediately, and stored at -80°C until further analysis.

Environmental data, samples used for microbial analysis and physiochemical analysis were collected simultaneously. The temperature and salinity of the seawater adjacent to seagrass samples (within 3 cm) was measured using a YSI 6600V2 water quality sonde (YSI, Yellow Springs, OH, United States). Dissolved oxygen (DO) concentrations and pH values were measured using a portable pH/DO Meter (Thermo Fisher Scientific, Inc., Beverly, MA, United States). Inorganic nutrients in seawater, including ammonium, nitrate, nitrite, and phosphate, were measured using standard methods as described previously (Huang et al., 2003). Nitrogen and carbon content of seagrass tissues (L and R) were determined according to Lee et al. (2004), and phosphorus content was analyzed by the colorimetric analysis of phosphate concentration (Fourqurean et al., 1992). Chemical data (Nitrate, ammonium and active phosphorous) of sediments were determined by using standard oceanographic methods (General Administration of Quality Supervision, Inspection and quarantine of the People’s Republic of China, 2002).

### DNA and RNA Extraction, cDNA Synthesis, PCR, Cloning, and Sequencing

DNA and RNA from approximately 1 g of sample (sediment or plant tissue; wet weight) were extracted using the E.Z.N.A^®^ Soil DNA kit and E.Z.N.A^®^ Soil RNA kit (Omega Bio-tek, Norcross, GA, United States) according to the manufacturer’s protocols. Synthesis of cDNA from extracted RNA was performed according to Li and Gu (2013). The nucleic acid concentrations were quantified using a NanoDrop 2000 spectrophotometer (Thermo Scientific, Wilmington, DE, United States). All qualified DNA and cDNA were stored at -80°C until analysis. For clone library analysis, archaeal and bacterial *amo*A gene sequences were amplified using the primer sets Arch-*amo*A F (5′-GGGGTTTCTACTGGTGGT-3′) and Arch-*amo*A R (5′-CCCCTCKGSAAAGCCTTCTTC-3′) (Francis et al., 2005) and *amo*A-1F (5′-STAATGGTCTGGCTTAGACG-3′) and *amo*A-2R (5′-GGGGTTTCTACTGGTGGT-3′) (Rotthauwe et al., 1997), respectively.

PCR reaction mixture for amplifying the *amo*A gene was prepared in accordance with details described by Hu et al. (2011). The PCR amplification conditions for *amo*A gene in AOA and AOB were in accordance with previously established protocols (Rotthauwe et al., 1997; Francis et al., 2005; Schmid et al., 2005; Li et al., 2011). The amplification was performed as follows: 5 min at 95°C, followed by 30 cycles of 45 s at 95°C, 60 s at 53°C and 60 s at 72°C, and 10 min at 72°C. For bacterial *amo*A gene amplification, the PCR conditions were 5 min at 95°C, followed by 30 cycles of 45 s at 95°C, 90 s at 56°C and 60 s at 72°C, and 10 min at 72°C. The PCR products from three reactions were pooled together to minimize PCR amplification bias, purified, and ligated into pMD18-T vector (TaKaRa, Dalian, China) according to the manufacturer’s instructions. Recombinant *Escherichia coli* cells were inoculated in Luria-Bertani broth containing ampicillin and incubated overnight at 37°C, and the plasmids carrying the target genes were extracted using a MiniBEST Plasmid Purification kit (TaKaRa, Dalian, China). Cloned *amo*A gene fragments were reamplified using primers M13-F (5′-AGGGTTTTCCCAG-TCACGACG-3′) and RV-R (5′-AGCGGATAACAATTTCACACAGG-3′). The target fragment sizes of archaeal and bacterial *amo*A genes were 491 and 635 bp, respectively. The PCR products were cloned into the pMD18-T vectors (TaKaRa, Dalian, China). PCR products were screened for the correct size and purity by 1% agarose gel electrophoresis, and clones showing the correct size were sequenced.

### Quantification of *amo*A Gene Copy Number at the DNA and Transcript Levels

Absolute quantification of archaeal and bacterial *amo*A genes were determined for both DNA and cDNA using qPCR in triplicate reactions with the LightCycler 480 System (Roche Diagnostics, Mannheim, Germany) and the following conditions: 95°C for 30 s, followed by 40 cycles of 30 s at 95°C, 60 s at 56°C, and 60 s at 72°C for archaeal *amo*A gene, or 95°C for 30 s, followed by 40 cycles of 30 s at 95°C, 60 s at 58°C, and 35 s at 72°C for bacterial *amo*A gene. To construct standard curves, archaeal and bacterial *amo*A genes were cloned into the pMD18-T vector (TaKaRa, Dalian, China) and then transformed into *E. coli* DH5*a*. The methods were similar to those used for the clone library construction. Recombinant plasmids carrying the target genes were extracted using a TaKaRaMini BEST Plasmid Purification Kit and quantified with a NanoDrop 2000 spectrophotometer. The copy numbers of *amo*A gene from the extracted plasmids were calculated by the concentrations and average base pairs of the plasmid. Standard curves for the archaeal *amo*A gene were constructed using standard plasmids obtained from the most dominant genotype clone ARSYTL81(KY794979), and for the bacterial *amo*A gene from the most dominant genotype clone BRNASYTRS53 (KY795002). Standard curves ranging from 10^3^ to 10^8^ gene copies/μL were obtained using 10-fold serial dilutions of linearized plasmid pMD-18T containing the cloned archaeal and bacterial *amo*A genes, respectively.

Real-time PCR efficiencies for AOA and AOB for DNA and cDNA were calculated according to *E* = 10 [-1/slope] (Rasmussen, 2001). The results showed that the amplification efficiencies ranged from 94.5 to 101%, with an R^2^ of standards higher than 0.99. The specificity of the amplification products was confirmed by melt-curve analysis, and the amplified fragments were checked by electrophoresis in 1.0% gel to confirm the expected sizes of amplicons. The size of archaeal and bacterial *amo*A genes were 491 and 635 bp, respectively. As the gene copies in the 1 μL of template DNA were determined, the final *amo*A gene and cDNA abundance of the seagrass and sediment samples were obtained by calculation. The results were expressed as gene copy abundance per gram of sediment or plant tissue (wet weight).

### Statistical Analysis

The obtained DNA and cDNA sequences were examined and checked for chimeras using the Check Chimera program of the Ribosomal Database Project (Cole et al., 2007). The operational taxonomic unit (OTU) reads were checked against a local *amo*A gene database (Ribosomal Database Project FunGene^1^) and the NCBI database^2^. Diversity indices were also evaluated using the MOTHUR program (Schloss et al., 2009; Li and Gu, 2013). Diversity statistics, including Shannon–Wiener (*H*′), Simpson (*D*) and species richness estimator (*S*Chao1), were calculated. Library coverage (C) was calculated as [1-(*n*/*N*)] × 100, where *n* is the number of OTUs represented by one clone (singletons) and *N* is the total number of sequences (Good, 1953). Diversity indices and richness estimators are useful statistical methods for comparing the relative complexity of AOA and AOB communities and for assessing the completeness of sample analysis. Reference sequences were selected by comparison with the GenBank database using BLAST, and the closest matches were included in the alignment and phylogenetic analysis with MEGA 6 (JCVI, Rockville, MD, United States) through neighbor-joining trees using Kimura 2-parameter distance with 1000 replicates to produce bootstrap values (Tamura et al., 2013).

One-way statistical analysis of variance (ANOVA) (confidence limit of 95%, *P* < 0.05) was performed to analyze variables among the three locations. In addition, one-way analysis of similarity (ANOSIM) was performed based on Bray-Curtis distances of AOA and AOB communities among the three sampling locations using the PRIMER v.6 software package (PRIMER-E, Plymouth, WA, United States) (Clark and Gorley, 2006). Moreover, Pearson’s correlation analysis of the abundance and diversity of AOA and AOB with the determined physicochemical parameters (water, tissue and sediment) was analyzed by SPSS v19.0 software (IBM, Inc., Chicago, IL, United States). Multi-Variate Statistical Package (MVSP, version 3.2, Kovach Computing Services, Anglesey) software was used to construct the similarity matrix and dendrograms (Kovach, 1999). In addition, genetic similarities among all clones were calculated by the percent similarity coefficient. Principal coordinate analysis (PCoA) was employed to depict the general ordination patterns of all samples at both DNA and transcript levels. In addition, the weighted pair-group method with arithmetic analysis (WPGMA) was used to generate similarity matrices and dendrograms by percent similarity using MVSP (Kovach, 1999).

### Nucleotide Sequence Accession Numbers

Representative sequences of archaeal and bacterial *amo*A genes for each OTU reported in this study have been deposited in the GenBank database under accession numbers MF796361–MF796384 and MF796347–MF796360, respectively.

## Results

### Environmental Parameters, Plant Tissues, and Sediment Characteristics of the Samples

The features of four different types of samples from the collection locations were analyzed, and the results are listed in Supplementary Table S1. The pH and salinity of all sampling locations ranged from 8.22–8.39 and 24.70–27.87‰, respectively. The highest DO concentration was recorded at AT, and the lowest at site SYT. The nitrate concentrations for the three locations ranged from 0.014 to 0.025 μM. The pH, salinity and DO concentration at SYT was significantly lower than at AT and ST, though the concentration of nitrite and ammonium were higher at SYT than at AT and ST (*P* < 0.05). Nonetheless, there was no significant difference in the nitrate concentrations among the three sampling sites (*P* > 0.05). The highest carbon content was found in the leaves from AT and the lowest value was recorded in the leaves from ST. The nitrogen content, phosphorus content and carbon percentage of roots and leaves from the three different locations were all significantly different (*P* < 0.05). The values of the bulk sediment environmental parameters (nitrate, ammonium, and active phosphorous concentrations) were all below the limit of detection. As illustrated in Supplementary Table S1, the ammonium and active phosphorous contents of the three RS samples ranged from 1.45 to 5.97 mg/kg and 14 to 15 mg/kg, respectively. Statistically, these three environmental parameters of AT, ST, and SYT rhizosphere sediments were significantly different (*P* < 0.05).

### Abundance of Archaeal and Bacterial *amo*A Genes

**FIGURE 1:**
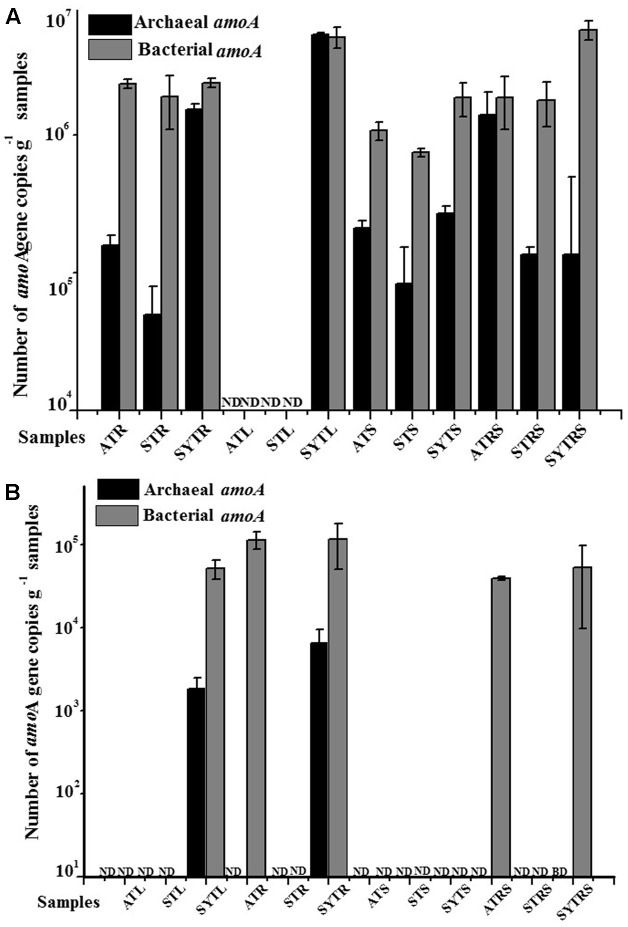
Abundance of the archaeal and bacterial *amo*A genes at the DNA **(A)** and cDNA **(B)** levels in the different samples. ND, not determined; BD, below limit of detection.

The abundances of AOA and AOB *amo*A genes at the DNA and transcript levels as determined by real-time PCR are shown in **Figure 1**. The abundance of all AOB communities (with the exception of that in sample SYTL) was higher than that of AOA at both the DNA and transcript levels. The abundance of AOA communities at the DNA level ranged from below the detection limit to 5.35 × 10^6^ gene copies per gram of sample (L, R, RS, and S); the abundance of AOB communities at the DNA level was between 7.68 × 10^5^ and 5.74 × 10^6^ gene copies per gram of sample (wet weight). In addition, the abundance of AOA communities at the transcript level was one order of magnitude lower than that at the DNA level. Their abundance at the DNA level ranged from below the detectable levels to 6.45 × 10^6^ copies g^-1^ per gram of sample, and the abundance of AOB communities had a wide range, from below the limit of detection to 1.15 × 10^6^ copies g^-1^ per gram of sample. Moreover, the ratio of AOB to AOA at the DNA level was highly variable in all samples. The highest value 42.21 was detected in the SYTRS sample (Supplementary Table S2). The ratio of DNA to cDNA for *amo*A gene copies for AOA community in sample SYT was greater than 20 and greater than 200 for sample SYTL. However, the values for AOB communities ranged only from 2.04 to 10.74 (Supplementary Table S2). When combing all the samples, the abundance of AOB communities was higher than that of AOA.

Bacterial *amo*A gene libraries from 13 samples (10 DNA-based and 3 cDNA-based) were successfully constructed. Overall, DNA sequences from 342 clones and cDNA sequences from 94 clones for archaea were recovered, while for bacterial *amo*A gene, there were 255 clones at the DNA level and 228 clones at the transcript level (Supplementary Tables S3, S4). Pearson’s correlation analysis revealed that the abundance of AOA at the DNA level in all RS showed significant positive relationships with the concentrations of seawater ammonium and the nitrogen contents of seagrass roots and leaves (*P* < 0.05), respectively. However, there was no such relationship between the environmental parameters and the abundance of AOB communities.

### Diversity of the Archaeal and Bacterial *amo*A Genes

**FIGURE 2:**
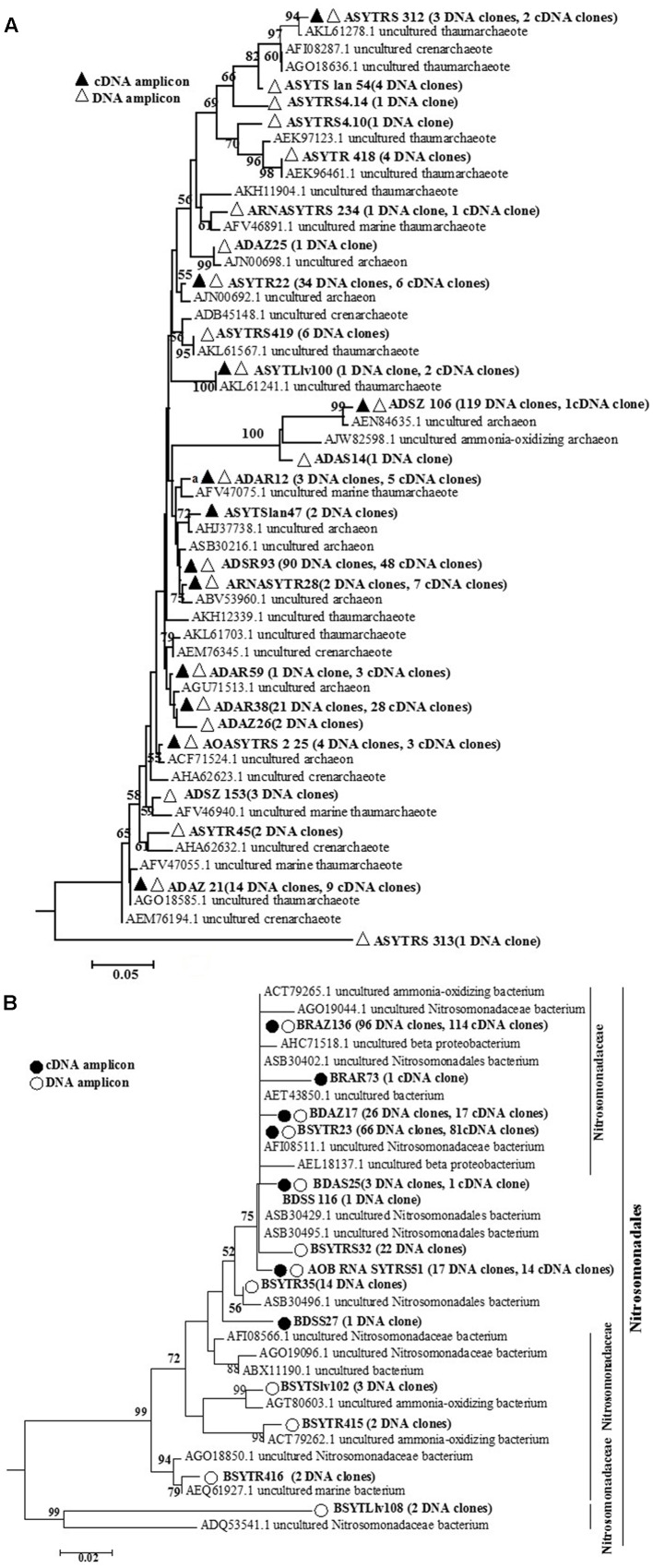
Phylogenetic tree constructed using distance and neighbor-joining method for archaeal *amo*A sequences **(A)** and bacterial *amo*A gene sequences **(B)** translated from cloned archaeal *amo*A and bacterial *amo*A gene sequences at the DNA and transcript levels, as recovered from the seagrass *Thalassia hemprichii* and their closest matches in GenBank from DNA samples and cDNA samples. Bootstrap values greater than 50% of 100 resamplings are shown near the nodes.

The number of sequenced clones differed among samples (ranging from 17 to 47), and they were then used to calculate diversity estimators (**Table 1**). The coverage, diversity, and richness indices of the nine cDNA-based libraries are summarized in **Table 1**. The coverage for AOA and AOB at the two different levels ranged from 72.73 to 100% and 94.59 to 100%, respectively (**Table 1**). Consequently, our results might have reflected the majority of archaeal and bacterial *amo*A gene clones at the DNA and transcript levels in our samples (**Figures 2A,B**).

**Table 1:** Biodiversity and predicted richness of the archaeal and the bacterial *amo*A gene sequences.

		AOA						AOB					
			
	Sample	Number of clones	Number of OTU	*C* (%)	*H′*	*D*	*S*_chao1_	Number of clones	Number of OTU	*C* (%)	*H′*	*D*	*S*_chao1_
DNA	SYTL	29	6	82.76	1.306	0.094	9.00	17	3	100	0.804	0.779	3.00
	SYTR	38	8	80.49	1.243	0.467	14.00	37	5	94.59	1.089	0.542	6.00
	SYTS	22	5	72.73	1.174	0.225	6.00	26	5	96.15	1.159	0.400	5.00
	SYTRS	45	11	80.85	2.047	0.170	17.00	41	4	97.56	1.18	0.859	4.00
	ATL												
	ATR	30	4	96.97	1.024	0.176	4.00						
	ATS	33	4	90.91	0.405	0.824	7.00	30	2	100	0.451	1.00	2.00
	ATRS	39	5	90	0.882	0.532	5.00	43	3	100	0.543	1.00	3.00
	STL												
	STR	47	1	97.87	0	0.957	1.00						
	STS	24	2	100	0.443	0.427	3.00	24	4	95.83	0.514	0.916	7.00
	STRS	37	3	94.59	0.640	0.506	3.00	37	2	97.3	0.124	0.946	2.00
	Total	**342**	**24**					**255**	**12**				
cDNA	SYTL	34	6	91.17	1.023	0.328	9.00	47	1	97.87	0	0.957	1.00
	SYTR	41	5	100	1.244	0.349	5.00	48	2	100	0.679	1.00	1.00
	SYTS												
	SYTRS	20	9	75	1.861	0.174	12.33	29	2	100	0.150	1.00	2.00
	ATL							34	2	100	0.630	0	2.00
	ATR							38	4	100	0.898	1.00	5.00
	ATS												
	ATRS							32	2	100	0.685	1.00	2.00
	STL												
	STR												
	STS												
	STRS												
	Total	**94**	**11**					**228**	**7**				


*OTUs of *amo*A sequences were generated by FunGene. C, coverage of the constructed clone libraries; *H′*, Shannon–Weiner index; D, Simpson index; *S*_*Chao1*_, richness estimators. SYT: *T. hemprichii* from Sanya Bay; AT: *T. hemprichii* from the airport area of Xisha Islands; ST: *T. hemprichii* from the stone Islands area of Xisha Islands; -L, -R, -S and -RS referred to leaf, root, bulk sediment and rhizosphere sediment, respectively; the total OTUs for the whole AOA and AOB communities were obtained by analyzing all the clones at the DNA level and transcript level, respectively.*

The biodiversity and richness indices of AOA and AOB communities at the DNA and cDNA levels are presented in **Table 1**. Rarefaction analyses were performed for all the bacterial or archaeal *amo*A gene clone libraries (Supplementary Figure S2). The highest diversity indices for AOB at the two levels were found for SYT. Overall, the indices indicated that AOB were less diverse than those for AOA, and the diversity indices at the DNA level were higher than those at the transcript level.

BLAST results indicated that over 80% of the obtained sequences recovered from this study were related to the sequences of marine sources, such as the marine water column, intertidal and marine sediments, mangrove sediments and marine sponges. The 24 archaeal *amo*A gene sequences share 87.50–99.48% sequence similarity with the closest GenBank matches. For bacterial *amo*A gene sequences, similarity of the 14 bacterial *amo*A genes ranged from 98.36 to 99.79%.

For AOA communities, 24 OTUs and 12 OTUs were found at the DNA and transcript levels, respectively, with 11 OTUs shared at both levels (**Figure 2** and **Table 1**). For AOB communities, the number decreased to 14 OTUs and 7 OTUs, with 6 OTUs shared at the two levels (**Figure 2** and **Table 1**). Based on the phylogenetic analysis, all bacterial *amo*A gene sequences (256 DNA sequences and 194 cDNA sequences) were mainly grouped into the *Nitrosomonadaceae* cluster (9 OTUs: 9 DNA sequences and 3 cDNA sequences) (**Figure 2B**).

The diversity indices showed no significant relationships between the values of *H*′ and *S*_Chao1_ with the concentrations of ammonium and phosphate (*P* > 0.05) for both AOA and AOB communities at the DNA level, respectively. However, our investigation revealed that there was a significant negative relationship between *S*_Chao1_ and the concentration of nitrite for AOB communities (*P* < 0.05).

### Variations in AOA and AOB Community Composition within and among Locations

For the archaeal *amo*A gene libraries, 1 to 11 and 5 to 9 OTUs at the DNA and transcript levels, respectively, were found for different samples. For AOB, only 2 to 5 and 1 to 2 OTUs were obtained for different clones at the DNA and transcript levels, respectively. Some OTUs could be found among almost all the samples, such as ADSR 93 for AOA at the DNA level, sharing approximately 99.37% similarity with an uncultured archaeon clone (KY357274) isolated from mangrove sediment. For AOB at the DNA level, the OTU BSYTR23 exhibited 99.79% similarity with the uncultured bacterial clone HaAOB1 (JN177536) retrieved from marine sponges. However, other OTUs were only detected in ATR, such as the OTU BRAZ73 at the transcript level. The BLAST result for OTU BRAZ73 indicated high similarity to a clone (KC893630) retrieved from the marine sponge *Spheciospongia vesparium.*

**FIGURE 3:**
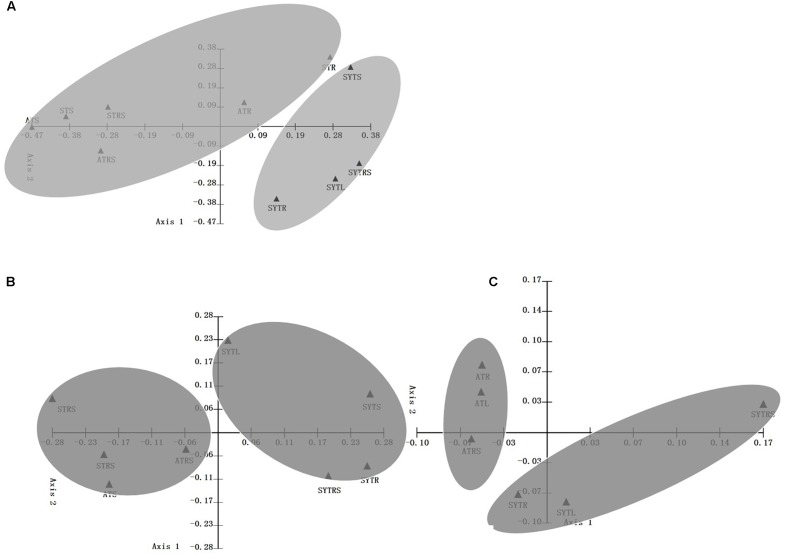
Principal coordinate analysis (PCoA) with a weighted UniFrac algorithm using archaeal and bacterial *amo*A gene sequences recovered from the seagrass *T. hemprichii* in coral reef ecosystems. Shown are the plots of the first two principal coordinate axes for PCoA and the distribution of AOA (**A**: DNA level) and AOB (**B**: DNA level; **C**: transcript level) (designated with the sampling station names) communities in response to these axes.

As shown in Supplementary Figure S3, samples obtained from the same location tended to group together regardless of the sample type. For instance, AT and ST samples first grouped with samples obtained from the same location and then grouped together, whereas, SYTS, SYTRS, and SYTR samples shared a high similarity of community composition (Supplementary Figure S3). For the DNA-based analysis, the first two principal coordinates (P1 and P2) could explain 70.40 and 76.77% of the total community variability in PCoA in archaeal and bacterial ammonia oxidizers, respectively (**Figures 3A,B**). The percentage of variability explained by the first two principal coordinates was 87.10% at the transcript level for AOB (**Figure 3C**). The WPGMA results were consistent with the PCoA plots (**Figure 3**). Consequently, the AOA and AOB communities shared higher similarity within the same location than within the same type of samples.

## Discussion

### AOA and AOB Abundance in Different Niches and Locations

All samples collected from the three locations were analyzed by DNA-based and transcript-based approaches. Most of the archaeal and bacterial *amo*A gene sequences at the DNA level in this study were successfully recovered, whereas a few samples at the transcript level were retrieved. This may be due to low gene copy number or expression of *amo*A gene in the relatively oligotrophic coral reef ecosystems, resulting in an abundance below the limit of detection. Compared with samples from AT and ST, archaeal and bacterial *amo*A gene sequences in most samples collected at SYT were successfully recovered. Previous investigations demonstrated that environmental factors, such as ammonia, temperature, salinity, dissolved oxygen and pH, had strong influences on the distribution of AOA and AOB (Li et al., 2011; Cao et al., 2015; Wang et al., 2015). For instance, Li and Gu (2013) showed that the diversity, abundance, and transcriptional activity of AOA and AOB shift in response to N conditions, specifically noting that ammonium amendment increased diversity and a lower nitrite concentration may reduce AOA and AOB diversity. Low temperature also exerted an important effect on the composition of AOA and AOB communities, which exhibited the lowest diversity when exposed to cold water (Urakawa et al., 2008). Different species respond differently to environmental variation, and the results of our investigation revealed the ammonium concentration to be a decisive factor for AOA and AOB community composition.

A stimulation experiment conducted by Prosser and Nicol (2012) suggested that AOA grew faster than AOB at lower ammonia concentrations, as the AOA affinity for ammonium was up to 200-fold that of AOB (Martens-Habbena et al., 2009, 2015). It has also been reported that AOA prefer to inhabit environments with lower ammonia concentrations and have higher *amo*A gene transcriptional activity than AOB in ammonia-limited water environments. In addition, Takano et al. (2010) discovered that deep-sea archaea adopted the strategy of recycling membrane lipids between growing cells and the surrounding sediment for saving energy to thrive in low ammonium habitats. However, in ammonia-rich areas, AOB communities would be the dominant component and contribute more to ammoxidation (Zhang Y. et al., 2015). Furthermore, plant species and their densities have crucial roles in determining community composition (Wang et al., 2015; Ettinger et al., 2017). Li et al. (2011) found that the presence of mangroves to increase the abundance of AOA and AOB in the mangrove sediment.

The qPCR quantification results presented in this study suggested that bacterial *amo*A gene abundance in almost all samples was higher than that of the archaeal *amo*A gene copy number with the exception of sample SYTL (**Figure 1**). The bacterial *amo*A gene abundance in the South China Sea was reported to range from 4.24 × 10^4^ to 1.99 × 10^6^ copies per gram of sediment (wet weight), which was consistent with our results (Cao et al., 2012). In addition, Dang et al. (2010a) found that the abundance of β-AOB was much higher than that of the archaeal *amo*A gene, and Wang et al. (2015) reported that the abundance of AOB at the transcript level was two orders of magnitude higher than that of AOA in a mangrove ecosystem. These findings were in agreement with our results. Furthermore, plants affected the bacterial community composition and activity by competing with rhizosphere microbes for nutrients, such as ammonium, nitrate, urea, and amino acids as nitrogen sources (Skiba et al., 2011). Foreseeably, microbes in rhizosphere sediment may utilize the low molecular weight compounds diffused from plant roots as carbon sources (Philippot et al., 2013). The ratio of β-AOB-*amo*A/archaeal *amo*A ranged from 212: 1 to 3090: 1 in deep-sea methane seep sediments of the Okhotsk Sea (Dang et al., 2010b). By comparison, the abundance ratio of AOA to AOB in our study was much lower, ranging from 0.96: 1 to 42.21: 1 (Supplementary Table S2).

### Diversity of Ammonia-Oxidizing Archaeal and Bacterial Communities

The clusters of archaeal *amo*A gene sequences obtained in this investigation were mainly from uncultured Thaumarchaeota originating from the marine environment. A chemolithoautotrophic marine crenarchaeote has been isolated, and its role in relation to nitrification has been shown to contribute significantly to global nitrogen and carbon cycles (Könneke et al., 2005). In addition, thaumarchaeotes have been found to play a crucial role in nitrification in both marine and terrestrial environments (Leininger et al., 2006; Wuchter et al., 2006; Beman et al., 2008; Erguder et al., 2009; Martens-Habbena et al., 2009). Moreover, Thaumarchaeota accounted for almost 12% of all archaeal sequences retrieved in Checker Reef sediments, and these organisms preferred oxic rather than anoxic sediments (Rusch et al., 2009; Gaidos et al., 2011; Pester et al., 2011). Beman et al. (2007) analyzed AOA communities associated with coral colonies from nine coral species and four different reef locations in the Gulf of California. Their results showed that *amo*A sequences were broadly distributed phylogenetically and that their closest relatives were related to sequences from coastal/estuarine sediments and oceanic water column sources. Conversely, they obtained no bacterial *amo*A gene sequences (Beman et al., 2007).

In our study, the most abundant OTU, ADSZ106 (119 clones at the DNA level and 1 clone at the transcript level), shared 93.96% similarity with uncultured archaeon clone S1–24 (KC758384) from saltwater aquaria. The bacterial *amo*A gene in our investigation was primarily affiliated with the cluster of *Nitrosomonadaceae* at the DNA and transcript levels (**Figures 2A,B**), accounting for 71.42% of all OTUs (**Figure 2B**). Many investigations have showed that most cultured AOB belonged to the family *Nitrosomonadaceae*, phylum Betaproteobacteria (Koops and Pommerening-Roser, 2001). Moreover, based on the species features, such as affinity for ammonia, and tolerance to salt and nitrite, microbes in this taxon could be further subgrouped into several clusters (Koops et al., 2006).

At the DNA level, the AOA communities were not significantly different between the sampling locations (*P* > 0.05), while for AOB communities, there were significant difference between SYT and AT (*P* < 0.05) and between AT and ST (*P* < 0.05) (**Table 2**). For all ammonia-oxidizing prokaryotes, some OTUs were universally present in all samples, whereas others occurred in only a few samples. For instance, some unique OTUs, e.g., ASYTRS419, ASYTRS312, and SYTRS313, were detected only in the samples collected at SYT. Zhao et al. (2012) reported many human activities, such as overfishing, reef rock digging and tourism activities in Sanya Bay, and all of these factors in combination with climate change have led to a significant decline in coral cover since the 1960s. The seagrass *T. hemprichii* gradually colonized under these environmental conditions at that location.

**Table 2:** One-way ANOSIM results for the AOA and AOB communities between the sampling locations Sanya Bay (SYT) and Yongxing Island (AT and ST) at the DNA level.

AOA DNA	Statistical value	*P*	AOB DNA	Statistical value	*P*
SYT vs. AT	0.556	0.057	SYT vs. AT	0.500	***0.013***
SYT vs. ST	0.500	0.086	SYT vs. ST	0.964	0.067
AT vs. ST	0.111	0.800	AT vs. ST	0.750	***0.033***


*Significant differences (*P* < 0.05) are indicated in italics and bold.*

### Higher Transcriptional Activity of AOB Other than AOA

A positive correlation between *amo*A gene copy numbers and the potential nitrification rate has been recorded (He et al., 2007). Consequently, quantitative assays targeting *amo*A gene transcripts were carried out in this study to analyze potential nitrification by AOA and AOB in coral reef ecosystems. The results showed that AOB would contribute more to the first step of nitrification for *T. hemprichii* (**Figure 1**). Furthermore, the results of an experiment conducted in the mangrove ecosystem were also consistent with our findings (Cao et al., 2015). However, Feng et al. (2016) obtained conflicting results for a marine sponge, for which the abundance of AOA was much higher than that of AOB at the cDNA level.

In our study, 24 OTUs and 14 OTUs were detected at the DNA level for AOA and AOB, respectively, and the number of OTUs decreased to 12 and 6, respectively, at the transcript level. This may be due to different preferences for ammonia. Hence, under the same condition, some species were dormant or below the PCR sensitivity threshold, whereas others exhibited high activity (Feng et al., 2016). For example, one unique bacterial *amo*A gene, OTU BDSS27, was detected only at the transcript level, and it was found to be related to uncultured ammonia-oxidizing bacterium clone ML-amoA-0 (FJ652557). A similar circumstance had been reported in the study of a marine sponge (Feng et al., 2016). This could be due to low abundance at the DNA level but high transcriptional activity. The most active *amo*A gene OTU in AOA communities was ADSR93, which was related to the uncultured archaeon clone GZ16110300849 (KY357274) obtained from mangrove sediments. Moreover, the most active bacterial *amo*A gene was the OTU BRAZ136 with 96 clones at the DNA level and 114 clones at the transcript level. Its closest BLAST hit was the uncultured bacterial clone HaAOB1 (JN177536) originating from the marine sponge *Haliclona* sp. collected from China East China at the depth of 20 m. Marine sponges are indispensable components of coral reef ecosystems and can help coral reefs thrive in ocean deserts because they absorb the nutrients from seawater and convert them into food for the reef and other marine organisms. Functional gene expression of ammonia-oxidizing microorganisms would also be altered by the health of their hosts. López-Legentil et al. (2010) reported that *amo*A gene expression was higher in fatally bleached sponges, whereas different patterns were observed in cyclic bleaching corals. Therefore, a higher abundance of AOB would have a more important function in transforming excess ammonia in the niche to maintain the healthy host.

## Conclusion

We herein described the abundance, diversity and transcriptional activity of the AOA and AOB communities of the seagrass *T. hemprichii* in three coral reef ecosystems at the DNA and cDNA levels. The diversity of AOA communities was higher than that of AOB, though the abundance of AOB communities was greater than that of AOB, and the community compositions of the sampling locations were distinct. As the focuses of this study were community composition and potential AOA and AOB activity, the amount of ammonia oxidized by AOA and AOB for the growth of seagrass, which is crucial for discerning the roles of ammonia-oxidizing microbes in ecosystems, was not determined. Consequently, the pattern for protein expression pattern of the *amo*A gene product and the ^15^N-isotope method would be used for elucidating their contributions to the seagrass productivity and their nitrogen transfer pathways for future investigations.

## Author Contributions

JL, LjL, and JD conceived the research. JL and XL performed the experiments. JL wrote the manuscript. YaZ and MA edited the manuscript. QY, LL, SZ, YiZ, and CW contributed to sampling or data analysis. All authors reviewed and approved the manuscript.

## Conflict of Interest Statement

The authors declare that the research was conducted in the absence of any commercial or financial relationships that could be construed as a potential conflict of interest.

## Footnotes

1 http://fungene.cme.msu.edu/index.spr

2 http://blast.ncbi.nlm.nih.gov/Blast.cgi
