# Supplementary material for: Community Composition and Transcriptional Activity of Ammonia-Oxidizing Prokaryotes of Seagrass Thalassia hemprichii in Coral Reef Ecosystems
Source: Front Microbiol. 2018 Jan 25;9:7. doi: 10.3389/fmicb.2018.00007 (PMC5788956; doi:10.3389/fmicb.2018.00007)
Supplement: Supplementary file 1 [file Data_Sheet_1.DOCX]

Supplementary Material

Community Composition and Transcriptional Activity of Ammonia Oxidizing Prokaryotes of Seagrass *Thalassia hemperichii* in Coral Reef Ecosytems

Juan Ling^1^, Xiancheng Lin^1,2,3^, Yanying Zhang^1^, Weiguo Zhou^1, 3^, Qingsong Yang^1, 3^, Liyun Lin^1, 3^, Siquan Zeng^1,3^, Ying Zhang ^1,2^, Cong Wang ^1,2,3^, Manzoor Ahmad^1, 3^, Lijuan Long^1^, Junde Dong^1,2 *^

*** Correspondence:**Junde Dong
[dongjd@scsio.ac.cn](mailto:dongjd@scsio.ac.cn)

# Supplementary Tables

**Table S1 Environmental parameters of water and characteristics of plant tissues as well as bulk and rhizosphere sediments**

| **Location** | **Water** | | | | | | | **Tissue** | | | | **Sediment** |  | | |
| --- | --- | --- | --- | --- | --- | --- | --- | --- | --- | --- | --- | --- | --- | --- | --- |
|  | **pH** | **Salinity** | **DO**  **(mg/L)** | **Nitrate**  **(mg/L)** | **Nitrite**  **(mg/L)** | **Ammonium**  **(mg/L)** | **Phosphate (mg/L)** |  | **Nitrogen （g/kg）** | **Phosphorus**  **（g/kg）** | **Carbon**  **(%)** |  | **Nitrate**  **(mg/kg)** | **Ammonium**  **（mg/kg）** | **Active phosphorous**  **（mg/kg）** |
| **AT** | 8.22  ±0.07 | 27.20  ±0.61 | 9.47  ±0.51 | 0.049  ±0.005 | 0.016  ±0.01 | 0.094  ±0.005 | 0.010  ±0.002 | **Leaf** | 10.00±0.12 | 1.30±0.21 | 27.40±0.02 | **Rhizosphere** | 44.32±1.05 | 4.37±0.085 | 14.30±0.706 |
|  |  |  |  |  |  |  |  | **Root** | 3.90±0.06 | 0.95±0.14 | 21.30±0.01 | **Bulk** | BD | BD | BD |
| **ST** | 8.39  ±0.10 | 27.87  ±1.86 | 7.78  ±0.72 | 0.058  ±0.008 | 0.011  ±0.001 | 0.063  ±0.05 | 0.016  ±0.007 | **Leaf** | 3.50±0.03 | 0.91±0.03 | 11.10±0.06 | **Rhizosphere** | 69.04±0.61 | 1.45±0.10 | 14.25±0.23 |
|  |  |  |  |  |  |  |  | **Root** | 3.00±0.05 | 0.96±0.41 | 14.50±0.01 | **Bulk** | BD | BD | BD |
| **SYT** | 8.15  ±0.042 | 24.70  ±0.90 | 6.70  ±0.59 | 0.044  ±0.007 | 0.025  ±0.02 | 0.121  ±0.007 | 0.015  ±0.001 | **Leaf** | 6.40±0.14 | 1.10±0.04 | 19.80±0.05 | **Rhizosphere** | 24.56±1.00 | 5.61±0.37 | 15.74±0.35 |
|  |  |  |  |  |  |  |  | **Root** | 4.50±0.20 | 1.20±0.08 | 28.10±0.42 | **Bulk** | BD | BD | BD |

BD: indicate below detection limit.

**Table S4 The ratio of AOB/AOA*amo*A gene copy at the DNA and transcript levels, and the ratio of DNA/cDNA *amo*A gene copy for AOA and AOB communities in all samples**

| **Samples** | **DNA**  **AOB/AOA** | **cDNA**  **AOB/AOA** | **AOA**  **DNA/ cDNA** | **AOB**  **DNA/ cDNA** |
| --- | --- | --- | --- | --- |
| **SYTS** | 6.94 |  |  |  |
| **SYTR** | 42.21 |  |  | 10.74 |
| **SYTL** | 0.96 | 27.84 | 287.14 | 9.88 |
| **SYTRS** | 1.55 | 17.89 | 23.75 | 2.06 |
| **ATS** | 5.10 |  |  |  |
| **ATRS** | 1.34 |  |  | 4.70 |
| **ATL** |  |  |  |  |
| **ATR** | 14.75 |  |  | 2.04 |
| **STS** | 8.94 |  |  |  |
| **STRS** | 13.02 |  |  |  |

**Table S2 Phylogenetic composition (based on OTUs) of AOA communities at the DNA and transcript level (number in the table indicates the clones of the OTU detected in the sample)**

|  | **DNA** | | | | | | | | | | **cDNA** | | | **Total** | **DNA** | **cDNA** |
| --- | --- | --- | --- | --- | --- | --- | --- | --- | --- | --- | --- | --- | --- | --- | --- | --- |
| **OTU** | **SYTL** | **SYTR** | **SYTRS** | **SYTS** | **ATR** | **ATRS** | **ATS** | **STR** | **STRS** | **STS** | **SYTL** | **SYTR** | **SYTRS** |  |  |  |
| **ADSZ106** | 1 | 3 | 1 | 0 | 6 | 30 | 30 | 0 | 26 | 22 | 1 | 0 | 0 | 120 | 119 | 1 |
| **ADSR93** | 5 | 1 | 16 | 10 | 4 | 1 | 1 | 46 | 4 | 2 | 21 | 22 | 5 | 138 | 90 | 48 |
| **ASYTR22** | 4 | 27 | 2 | 0 | 0 | 0 | 0 | 0 | 1 | 0 | 0 | 4 | 2 | 40 | 34 | 6 |
| **ASYTR418** | 1 | 1 | 1 | 1 | 0 | 0 | 0 | 0 | 0 | 0 | 0 | 0 | 0 | 4 | 4 | 0 |
| **ADAZ21** | 4 | 1 | 6 | 0 | 0 | 3 | 0 | 0 | 0 | 0 | 4 | 2 | 3 | 23 | 14 | 9 |
| **ADSZ153** | 0 | 0 | 0 | 0 | 0 | 1 | 1 | 0 | 1 | 0 | 0 | 0 | 0 | 3 | 3 | 0 |
| **ARNASYTL28** | 0 | 0 | 2 | 0 | 0 | 0 | 0 | 0 | 0 | 0 | 3 | 3 | 1 | 9 | 2 | 7 |
| **ADAR38** | 9 | 2 | 6 | 3 | 11 | 1 | 0 | 1 | 0 | 0 | 3 | 10 | 3 | 49 | 33 | 16 |
| **ASYTRS225** | 0 | 0 | 3 | 0 | 2 | 0 | 0 | 0 | 0 | 0 | 0 | 0 | 2 | 7 | 5 | 2 |
| **ASYTRS312** | 1 | 0 | 3 | 0 | 0 | 0 | 0 | 0 | 0 | 0 | 0 | 0 | 1 | 5 | 4 | 1 |
| **ASYTSlan54** | 0 | 0 | 0 | 4 | 0 | 0 | 0 | 0 | 0 | 0 | 0 | 0 | 0 | 4 | 4 | 0 |
| **ADAR12** | 0 | 0 | 0 | 1 | 5 | 0 | 0 | 0 | 2 | 0 | 0 | 0 | 0 | 8 | 8 | 0 |
| **ASYTLlv100** | 1 | 0 | 0 | 0 | 0 | 0 | 0 | 0 | 0 | 0 | 1 | 0 | 1 | 3 | 1 | 2 |
| **ASYTRS419** | 3 | 1 | 2 | 0 | 0 | 0 | 0 | 0 | 0 | 0 | 0 | 0 | 0 | 6 | 6 | 0 |
| **ADAZ26** | 0 | 0 | 0 | 0 | 0 | 2 | 0 | 0 | 0 | 0 | 0 | 0 | 0 | 2 | 2 | 0 |
| **ADAR59** | 0 | 0 | 0 | 1 | 2 | 0 | 0 | 0 | 0 | 0 | 0 | 0 | 1 | 4 | 3 | 1 |
| **ASYTSlan47** | 0 | 0 | 0 | 1 | 0 | 0 | 0 | 0 | 1 | 0 | 0 | 0 | 0 | 2 | 2 | 0 |
| **ASYTRS414** | 0 | 0 | 1 | 0 | 0 | 0 | 0 | 0 | 0 | 0 | 0 | 0 | 0 | 1 | 1 | 0 |
| **ADAZ25** | 0 | 0 | 0 | 0 | 0 | 1 | 0 | 0 | 0 | 0 | 0 | 0 | 0 | 1 | 1 | 0 |
| **ARNASYTRS234** | 0 | 0 | 0 | 1 | 0 | 0 | 0 | 0 | 0 | 0 | 0 | 0 | 1 | 2 | 1 | 1 |
| **ASYTRS313** | 0 | 0 | 1 | 0 | 0 | 0 | 0 | 0 | 0 | 0 | 0 | 0 | 0 | 1 | 1 | 0 |
| **ASYTR45** | 0 | 2 | 0 | 0 | 0 | 0 | 0 | 0 | 0 | 0 | 0 | 0 | 0 | 2 | 2 | 0 |
| **ADAS14** | 0 | 0 | 0 | 0 | 0 | 0 | 1 | 0 | 0 | 0 | 0 | 0 | 0 | 1 | 1 | 0 |
| **ASYTRS410** | 0 | 0 | 1 | 0 | 0 | 0 | 0 | 0 | 0 | 0 | 0 | 0 | 0 | 1 | 1 | 0 |
| **Total** | **29** | **38** | **45** | **22** | **30** | **39** | **33** | **47** | **35** | **24** | **33** | **41** | **20** | **436** | **342** | **94** |

**Table S3 Phylogenetic composition (based on OTUs) of AOB communities at the DNA and transcript levels (number in the table indicates the clones of the OTU detected in the sample)**

|  | **DNA** | | | | | | | | **cDNA** | | | | | | **Total** | **DNA** | **cDNA** |
| --- | --- | --- | --- | --- | --- | --- | --- | --- | --- | --- | --- | --- | --- | --- | --- | --- | --- |
| **OTU** | SYTL | SYTR | SYTRS | SYTS | ATRS | ATS | STRS | STS | SYTL | SYTR | SYTRS | ATR | ATRS | ATL |  |  |  |
| **BSYTR23** | 2 | 4 | 11 | 6 | 12 | 5 | 17 | 9 | 15 | 18 | 8 | 12 | 16 | 12 | 147 | 66 | 8 |
| **BRNASYTRS51** | 2 | 2 | 2 | 2 | 2 | 4 | 2 | 1 | 2 | 3 | 2 | 2 | 2 | 3 | 31 | 17 | 14 |
| **BRAZ136** | 7 | 16 | 18 | 8 | 17 | 15 | 10 | 5 | 29 | 25 | 18 | 17 | 11 | 14 | 210 | 96 | 114 |
| **BDAZ17** | 1 | 0 | 0 | 0 | 7 | 4 | 7 | 7 | 1 | 2 | 0 | 6 | 3 | 5 | 43 | 26 | 17 |
| **BSYTR35** | 0 | 9 | 3 | 2 | 0 | 0 | 0 | 0 | 0 | 0 | 0 | 0 | 0 | 0 | 14 | 14 | 0 |
| **BDAS25** | 0 | 0 | 0 | 0 | 1 | 2 | 0 | 0 | 0 | 0 | 1 | 0 | 0 | 0 | 4 | 3 | 1 |
| **BSYTRS32** | 3 | 4 | 7 | 4 | 4 | 0 | 0 | 0 | 0 | 0 | 0 | 0 | 0 | 0 | 22 | 22 | 0 |
| **BSYTR416** | 0 | 1 | 0 | 1 | 0 | 0 | 0 | 0 | 0 | 0 | 0 | 0 | 0 | 0 | 2 | 2 | 0 |
| **BSYTSlv102** | 0 | 0 | 0 | 3 | 0 | 0 | 0 | 0 | 0 | 0 | 0 | 0 | 0 | 0 | 3 | 3 | 0 |
| **BDSS116** | 0 | 0 | 0 | 0 | 0 | 0 | 0 | 1 | 0 | 0 | 0 | 0 | 0 | 0 | 1 | 1 | 0 |
| **BRAR73** | 0 | 0 | 0 | 0 | 0 | 0 | 0 | 0 | 0 | 0 | 0 | 1 | 0 | 0 | 1 | 0 | 1 |
| **BSYTR415** | 0 | 1 | 0 | 0 | 0 | 0 | 1 | 0 | 0 | 0 | 0 | 0 | 0 | 0 | 2 | 2 | 0 |
| **BSYTLlv108** | 2 | 0 | 0 | 0 | 0 | 0 | 0 | 0 | 0 | 0 | 0 | 0 | 0 | 0 | 2 | 2 | 0 |
| **BDSS27** | 0 | 0 | 0 | 0 | 0 | 0 | 0 | 1 | 0 | 0 | 0 | 0 | 0 | 0 | 1 | 1 | 0 |
| **Total** | **17** | **37** | **41** | **26** | **43** | **30** | **37** | **24** | **47** | **48** | **29** | **38** | **32** | **34** | **483** | **255** | **228** |

# Supplementary Figures

**
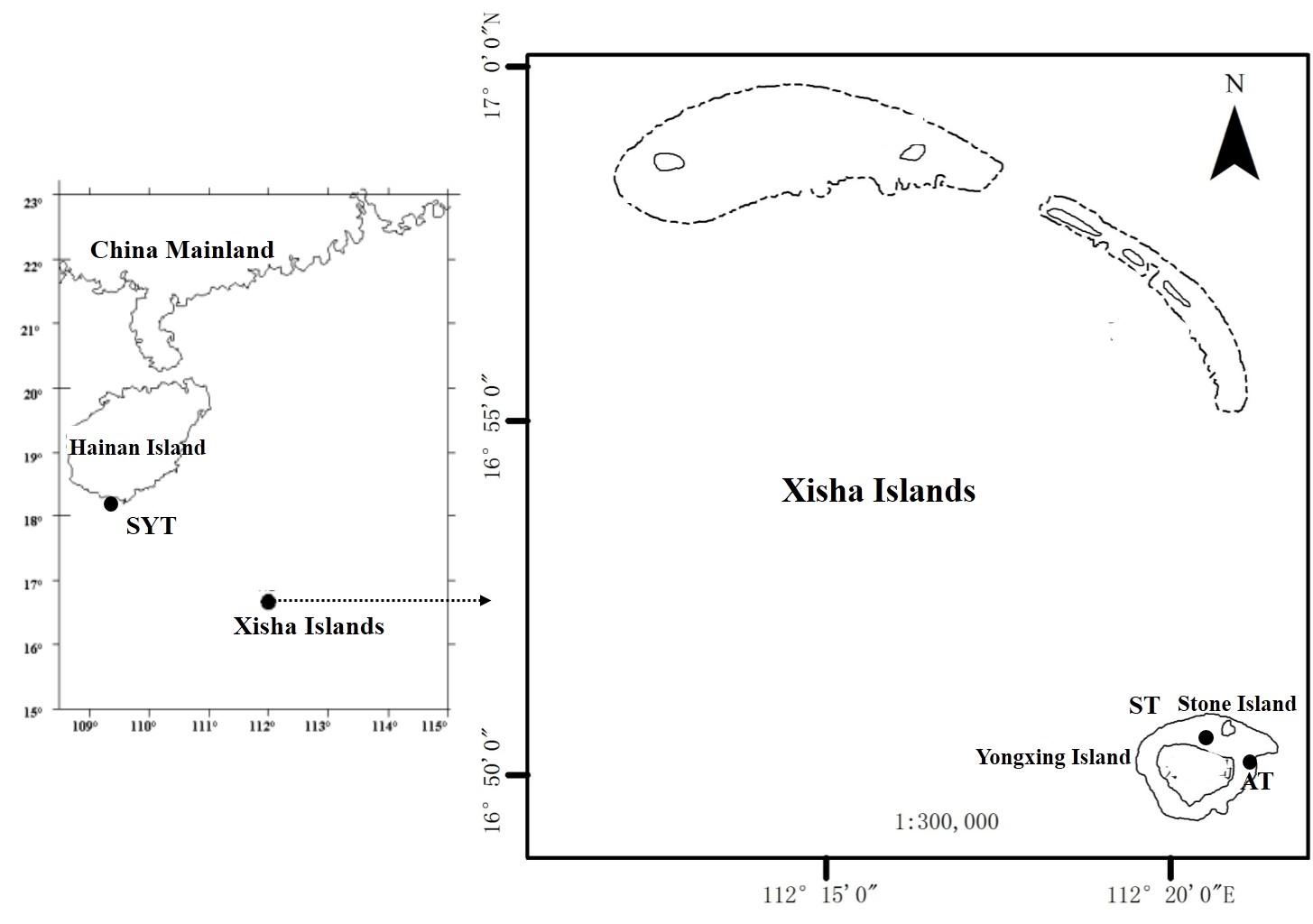
**

**Supplementary Figure S1.** Map showing the locations of the study sites in the Luhuitou fringing reef (SYT), Sanya Bay and Yongxing Island (ST and AT), Xisha Islands.


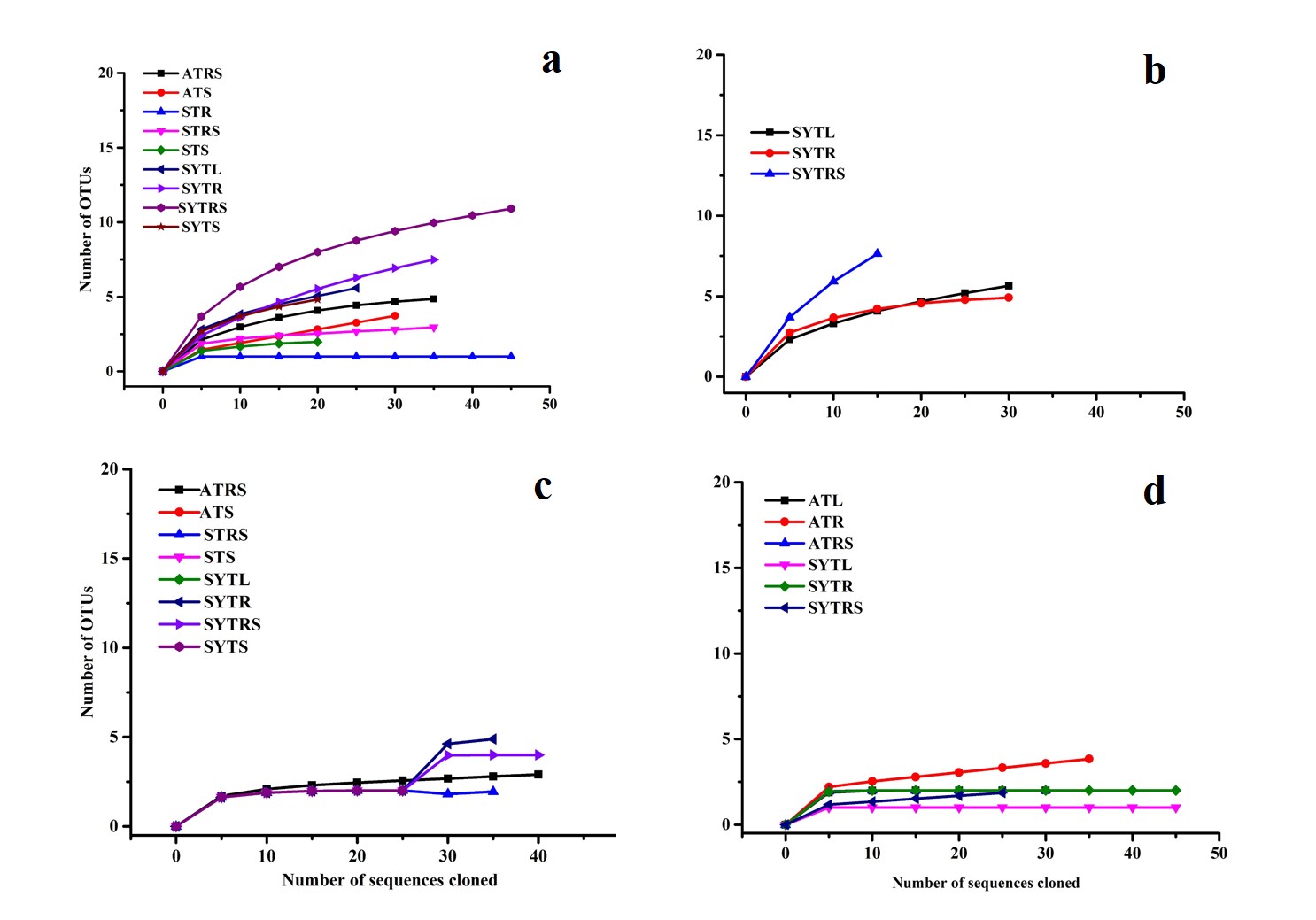


**Supplementary Figure S2**. Rarefaction curve analysis of archaeal *amo*A DNA (a) and cDNA (b) bacterial *amo*A DNA (c) and cDNA (d) gene clone libraries. Sample color codes are presented in the legend.


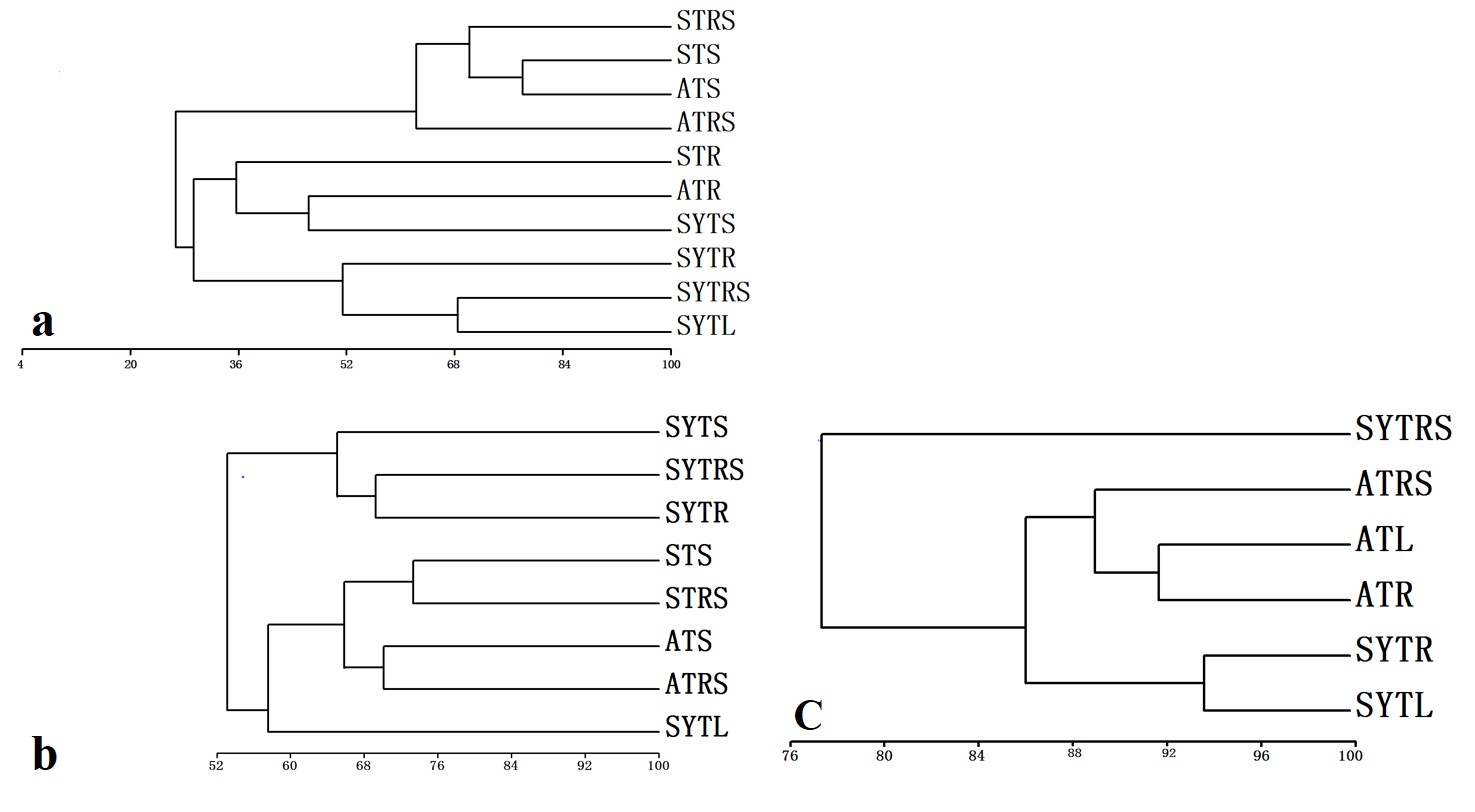


**Supplementary Figure S3.** Dendrogram generated by WPGMA cluster analysis of community composition (based on OTUs and their clones) based on percent similarity: AOA *amo*A DNA (a), AOB *amo*A DNA (b) and cDNA (c).
